# Physical Load Affects Perceptual-Cognitive Performance of Skilled Athletes: a Systematic Review

**DOI:** 10.1186/s40798-016-0061-0

**Published:** 2016-09-12

**Authors:** M. Schapschröer, S. Lemez, J. Baker, J. Schorer

**Affiliations:** 1 Institute of Sport Science, Carl von Ossietzky University Oldenburg, Ammerländer Heerstraße 114-118, 26129 Oldenburg, Germany; 2 School of Kinesiology and Health Science, York University, Toronto, Canada

**Keywords:** Expertise, Acute physical exercise, Perception, Cognition, Athlete

## Abstract

**Background:**

Many researchers have considered the impact of physical exercise on perceptual-cognitive performance. There have also been a substantial number of studies that have examined how perceptual-cognitive skills differ between elite athletes and non-athletes. However, the knowledge on how physical exercise interacts with perceptual-cognitive skill is limited.

This systematic review aims to provide detailed information on how athletes’ perceptual-cognitive performance is influenced by acute physical exercise load and whether these effects differ between elite athletes and lesser skilled groups.

**Methods:**

A systematic review was conducted using different combinations of the keywords *physical load*, *acute*, *exercise*, *perception*, *cognition*, *perceptual*, *cognitive*, *sport*, and *athlete* with the PubMed and SportDiscus databases. Additional articles were found through screening the references of these papers.

Articles had to (a) be full journal articles written in English, (b) include an athlete sample, (c) examine *acute effects* of physical exercise, and (d) measure a perceptual-cognitive task as the dependent variable.

**Results:**

Twenty-six articles matched the inclusion criteria. Results suggested the impact of acute physical exercise on perceptual-cognitive performances of athletes depends on the specificity of the induced exercise and perceptual-cognitive task. Additionally, speed and accuracy were influenced differently by physical exercise. Furthermore, skilled athletes seem to be more positively influenced by acute physical exercise than novices.

**Conclusion:**

Since many factors influence perceptual-cognitive expertise, future research should be highly precise (e.g., regarding the definition of variables, the intensity of the physical exercise) and specific (e.g., regarding the tasks used, the type of the physical exercise).

## Key Points

1. The impact of acute physical exercise on perceptual-cognitive performances of athletes depends on the specificity of the induced exercise and perceptual-cognitive task.
2. Acute physical exercise seems to improve expert athletes’ speed in perceptual-cognitive tasks but does not affect accuracy.
3. Expert athletes are more positively influenced by acute physical exercise than lesser skilled people.

## Background

Many researchers have considered the impact of physical exercise on perceptual-cognitive performance. For example, several reviews and meta-analyses have examined the influence of physical load on different aspects of cognition (e.g., [1–6]) or perception-action coupling (e.g., [7]). There has also been a substantial number of studies examining how perceptual-cognitive skills differ between elite athletes and non-athletes (for an overview, see [8]). However, our knowledge of how acute physical exercise influences perceptual-cognitive performance, particularly among performers at different skill levels, is limited and the results are controversial.

The impact of physical exercise on perceptual-cognitive performance is, among several other factors (e.g., the type of the cognitive task induced), dependent on the type (e.g., intensity, duration) of the induced physical exercise. In his review of the effects of acute exercise on cognition, Tomporowski [6] concluded that submaximal exercise of up to 60 min in duration facilitates information processing, but extended exercise impairs information processing and memory functions. Prior research suggests an inverted U-relationship (arguably caused by arousal), with moderate physical exercise intensity having a positive effect on perceptual-cognitive performance, while high exercise intensity has a negative effect [9]. However, the assumption of an inverted U-effect [9] has been criticized with several studies not supporting this hypothesis (e.g., [3, 4, 7]). Instead, it appears that different components, such as exercise intensity, duration, or task type interact and add complexity to our understanding of the impact of physical exercise on perception/cognition [3, 4, 10]. For example, Roig and colleagues [5] conducted a meta-analysis that investigated acute (29 studies) and long-term (21 studies) effects of cardiovascular exercise on human memory. The results from their review revealed that acute exercise had moderate-sized effects on short-term memory and moderate to large-sized effects on long-term memory [5]. Further, as it relates to speed and accuracy of cognition, McMorris et al. [3] revealed that acute, intermediate intensity exercise improves speed of response in working memory tasks but impairs accuracy, while no speed-accuracy trade-off was found. In another meta-analysis examining effects of differing intensities of acute exercise on cognition, McMorris and Hale [4] showed that speed and accuracy were affected differently; moderate intensity exercise produced faster speeds of processing without a significant change in accuracy. However, the authors suggested previous work may have failed to choose tests with sufficient complexity to elicit exercise-induced impacts on accuracy.

The complexity of the task is another factor that seems to be important when considering the impact of physical exercise on perceptual-cognitive performances. As McMorris and Hale [4] emphasized, complex tasks seem to be more affected by physical exercise than simple tasks. For speed, large effects of physical exercise on executive tasks were found while only small effects were shown for simple tasks [4]. For accuracy, no effects were found, regardless of the complexity of the task. In their recent meta-analysis, McMorris et al. [10] looked at the impact of physical exercise on cognition from a neurochemical perspective. Depending on the interaction of exercise, brain catecholamines (which are responsible for increases in arousal by activating the reticular formation) and cognition, McMorris et al. [10] describe three different types of tasks, differentiating between working memory tasks (e.g., Stroop color test, Simon task, switch visual attention tasks, tasks that require selecting relevant sensory information), attention/perception tasks (e.g., simple or choice reaction-time tasks with flashing lights, visual search tasks), and long-term memory tasks [3, 4, 10]. These three types of tasks seem to be differentially affected by physical exercise, which might be explained with the concentration and proportion of dopamine and adrenalin in the brain under different stress levels. While working memory tasks improve under moderate physical exercise, research indicates deterioration in these tasks during heavy exercise [10]. In contrast, attention/perception tasks show a linear improvement with increases in exercise intensity [10]. Finally, long-term memory or learning tasks seem to be especially positively influenced by heavy exercise [10].

Research investigating perceptual-cognitive expertise differences has overwhelmingly shown that experts outperform novices in several perceptual-cognitive areas, such as attention, processing speed or pattern recall and recognition tasks (e.g., [8, 11, 12]). When examining whether general or sport-specific performances differentiate between experts and non-experts, two different approaches have been examined. First, the “cognitive component skill approach” focuses on examining whether general cognitive measures capture expertise differences in sport (e.g., [12, 13]). Some authors argue that sport training (and the experiences gained with this) is a form of cognitive training that leads to, for example, more efficient brain networks and brain plasticity, which leads to enhanced cognitive processing [12]. Furthermore, some studies have shown that a high fitness level improves cognitive functioning because of changes to structural and functional aspects of the brain [13]. This is in line with the “Cardiovascular Fitness Hypothesis” that indicates regular exercise changes brain functions, which lead to cognitive benefits (e.g., [13]). In their meta-analyses on the relationship between expertise in sport in laboratory measurements, Voss et al. [12] noted that athletes are better in general processing speed and attentional tasks. However, the authors emphasized that the results are controversial and that the “cognitive component skill approach” has often been criticized for not considering the complex environment that might also be important when examining superior expert performances in sport [12, 14]. An alternative perspective, the “expert performance approach” aims to study athletes under ecologically valid and sport-specific contexts. This approach uses tasks that are representative for the specific domain of expertise and is therefore important when examining expertise differences in sport-specific perceptual-cognitive tasks [8, 11, 14]. In this context, the relatively high number of studies examining perceptual-cognitive expertise differences in sport reinforces the conclusion that skilled performers process information within their domain of expertise differently than novices. For example, Mann and colleagues’ [8] meta-analysis on perceptual-cognitive expertise in sport concluded that experts’ performances are superior for specific measurements of response accuracy and response time, which relates to better detection and processing of perceptual cues. Furthermore, differences were found in visual search behaviors, with experts having fewer fixations of longer duration compared to non-experts [8]. However, Mann et al. [8] indicated that certain factors, such as sport type or the type of the stimulus presentation, moderated the relationship between perceptual-cognitive skills and level of expertise. With these moderators in mind, and considering the specificity of perceptual-cognitive expertise (e.g., [15, 16]), the very small number of studies looking at the influence of different physical exercises on athletes’ perceptual-cognitive performances is surprising since athletes have to combine these tasks with the specific physiological demands of their sport.

### Objectives

The aim of this systematic review was to provide an overview of the existing literature on the impact of different acute physical exercises on perceptual-cognitive performances of athletes. In the context of many sports, different perceptual-cognitive skills (e.g., decision-making and anticipation performance or pattern recall and pattern recognition tasks (for an overview, see [8])) play important roles and have often been used to compare athletes to non-athletes. As noted earlier, two different approaches have been applied when examining experts’ superiority in perceptual-cognitive tasks and results have often been controversial (e.g., [12]). However, research has overwhelmingly shown that experts are superior to novices if structured and sport-specific tasks are used [8]. Considering (a) the relatively high number of studies examining the impact of physical load in non-athletes and (b) the specificity of perceptual-cognitive expertise, this review aims to provide detailed information on how athletes’ perceptual-cognitive performance is influenced by acute physical exercise load and whether these effects differ between elite athletes and lesser skilled groups.

## Methods

A systematic search was conducted following the guidelines from the Preferred Reporting Items for Systematic Reviews and Meta-Analyses (PRISMA) statement for systematic review [17].

### Information Sources and Search Process

The systematic search was performed using a general search engine (i.e., PubMed) and a sport-specific (i.e., SportDiscus) database. Nine combinations of keywords were included in both databases: “*Physical load + perception + sport*,” “*Physical load + cognition + sport*,” “*Acute + exercise + perception + sport*,” “*Acute + exercise + cognition + sport*,” “*Physical load + perception + athlete*,” *Physical load + cognition + athlete*,” “*Acute + exercise + perception + athlete*,” “*Acute + exercise + cognition + athlete*,” “*Exercise + perceptual + cognitive*.” The “All Fields” search query was utilized to provide the best opportunity to capture all relevant articles for our review. The article search was completed on December 15, 2015, and a publication year cut-off was not instituted. Additionally, the references from the articles found through the database searches were screened to filter out further articles that matched the inclusion criteria.

### Eligibility Criteria and Study Selection

The following inclusion criteria were instituted for this review: full journal articles (no abstracts), written in English, athlete participants, and the study had to measure a perceptual-cognitive-task as the dependent variable. Studies were included if they examined at least one group of athletes. More specifically, studies that examined “novices” and “non-athletes” but compared them to an athlete group were included in our review. Therefore, all levels of skill were included; however, studies that only looked at novices or non-athletes (i.e., no athletes tested) were excluded. The title and abstract of each article identified through the database search were screened to see whether the article matched the inclusion criteria. All studies looking at diseases or injuries as well as perceived exertion were excluded. Since this review focused on outcomes measured through performing a perceptual-cognitive task, neuroscientific studies (e.g., directly measuring brain functioning via EEG and “only” presenting data from neuroscientific measurements) were also excluded. Finally, only studies looking at *acute* effects of physical exercise on perceptual-cognitive performances were considered for the review.

### Data Items

Articles were analyzed by several criteria to build categories and synthesize the results. Generally, articles were screened with special consideration of the perceptual-cognitive task tested, the physical exercise induced, the time of testing, and the participants tested. To compare and summarize results, definitions of terms were established for each of the categories. Furthermore, the methodological quality of each study was evaluated by using the Mixed Methods Appraisal Tool (MMAT), in which the score can be presented using the descriptors *, **, ***, and **** depending on the number of criteria met out of four criteria that were evaluated per category/study [18].

#### Perceptual-Cognitive Task

Because of the importance of specificity in the context of perceptual-cognitive expertise (e.g., [15, 16]), the type of perceptual-cognitive task was classified as either “general” or “specific.” More concretely, a general perceptual-cognitive task was a task that was not specific to the participants’ domain of expertise (e.g., general reaction-time tasks without a sport-specific context). Correspondingly, a specific perceptual-cognitive task was one that was specific to the participants’ domain of expertise. Tasks were classified as being specific if they included either testing in real-game situations or under contexts similar to those of real games/competitions (e.g., soccer-specific choice reaction-time tasks using videos of game situations to test soccer players). Assuming that speed and accuracy might be affected differently by acute physical exercise (e.g., [4]), studies were also classified by what performance outcomes they measured in the task.

Since the complexity of the tasks also plays an important role when examining the influence of physical exercise on perception/cognition, the perceptual-cognitive tasks were also classified as either being a “working memory task” or a “attention/perception task” [10]. Because this systematic review focused on the impact of *acute* physical exercise, the third category “long-term memory task” was not tested in the studies included in this review.

#### Physical Exercise

Since the impact of physical exercise on cognitive functioning is dependent on the characteristics of the physical exercise load (e.g., [1, 19]), and considering the specificity of perceptual-cognitive expertise [15], physical exercises were also classified as general or specific. In accordance with the descriptions for the perceptual-cognitive task, a general physical exercise was an exercise that was not specific for the participants’ domain of expertise (e.g., exercises on a cycle ergometer for soccer players), whereas an example of a specific physical exercise includes an intermittent running protocol for soccer players.

In addition to the differences between these types of acute physical exercise, intensity also plays an important role in perceptual-cognitive performance (e.g., [20]). Therefore, we further classified studies by the intensity of the physical exercise induced. In line with the procedure used by McMorris and Hale [4] in their meta-analysis on the effects of differing intensities of acute exercise on different aspects of cognition, and based on the classification from Borer [21], we distinguished “low,” “moderate,” and “high” intensities. Low intensity exercise was defined as <40 % of maximum power output (*W*_max_), moderate intensity as between 40 and 79 % *W*_max_, and high intensity as ≥80 % *W*_max_. If studies did not report values for *W*_max_ but for maximum oxygen uptake (VO_2max_) or maximum heart rate (HR_max_), these values were converted by using the formulae from Arts and Kuipers [22]: VO_2max_ = 12.1 + 0.866 × %*W*_max_, %HR_max_ = 46.3 + 0.545 × %*W*_max_. This is the same procedure that McMorris and Hale [4] applied. For other given values of intensity (e.g., heart rate reserve), physical exercise was compared to the studies that provided data on *W*_max_, VO_2max_, or HR_max_ and were accordingly classified into one of the groups. In addition to these three classifications of exercise intensity, a fourth, “intermittent/interval exercise,” was added, which alternated high- and low-intensity exercises.

#### Participants

To create consistent terms for the different levels of expertise, participants were classified into three comparable groups: experts, advanced, and novices. Athletes were classified as “experts” if they were highly engaged in their sports and competed at a national level or higher. The term “elite athletes” is understood as being part of this expert group. “Advanced athletes” were performers who trained regularly, in a structured way, and competed at a state, provincial, or regional level. The term athlete therefore covers all participants who trained and competed in sports at least on a regional level, regardless of the exact competition level. In the classifications created for this systematic review, the term athlete therefore covers expert as well as advanced participants. Some studies also included participants without or with very little experience in the specific sport, meaning that they did not regularly train or compete in a structured way or that they never participated in sport. These participants are classified as “novices.”

### Risk of Bias

All studies identified through the database search were assessed by three experts in sport expertise research. Complete agreement regarding the eligibility of each study was necessary in order for it to be included in this review. Differences between assessors were discussed until unanimity was reached. Furthermore, the methodological quality of each individual study was evaluated by using the MMAT [18].

## Results

### Literature Search

**Fig. 1:**
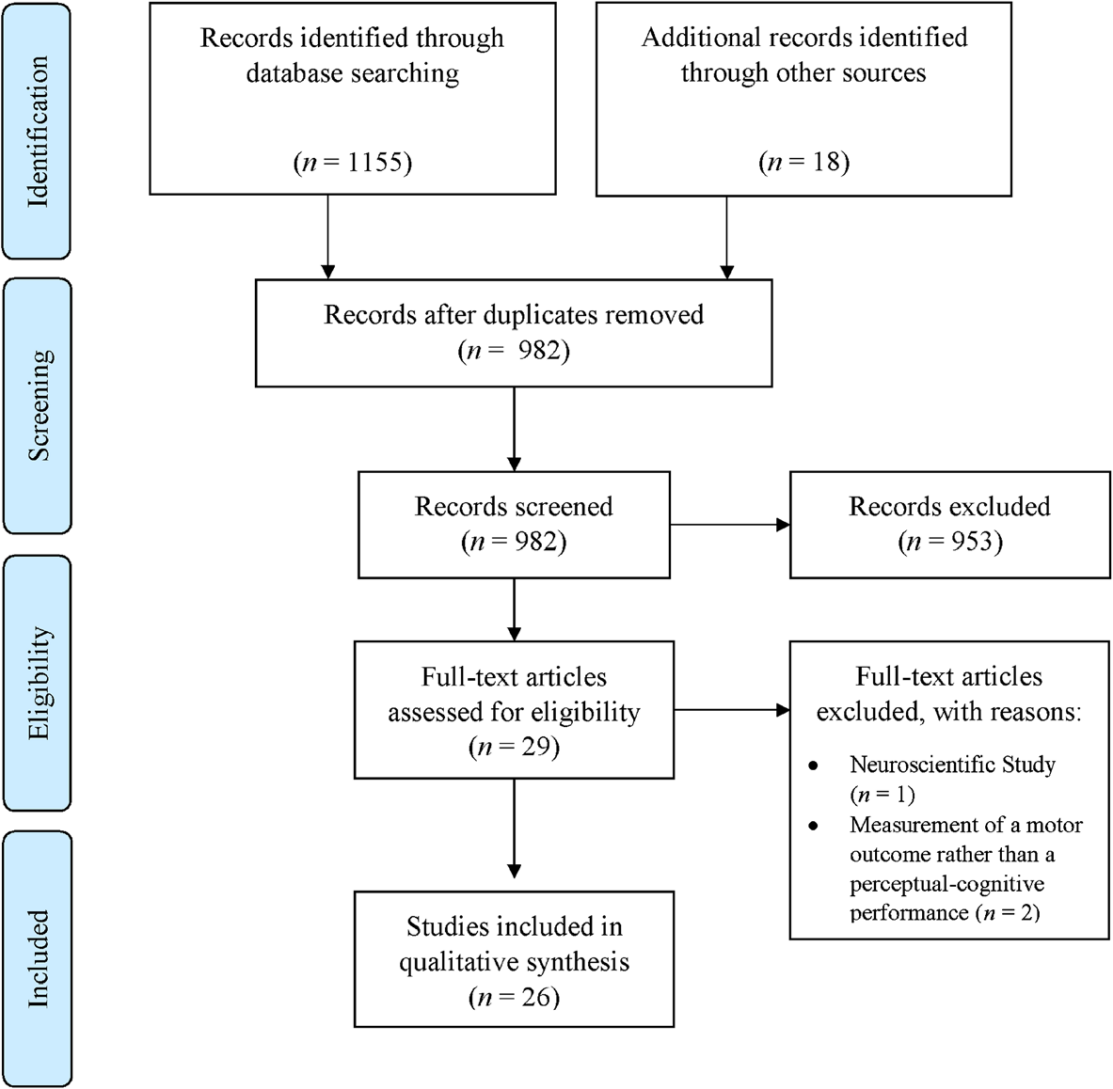
PRISMA 2009 flow diagram—overview of the complete selection process. From Moher et al. [17]

A total of 1155 articles were located through the systematic search, from which 191 duplicates were removed. An additional 18 articles were identified through searching the reference lists of the articles found through the database searches (meta-analyses and articles that matched the inclusion criteria) or through experts’ suggestions. Therefore, 982 articles were screened, from which 953 were excluded after screening the title and abstract because they did not match the inclusion criteria. The main reasons for exclusion were “physical load was not induced or was not the independent variable,” “perceptual-cognitive performance was not the dependent variable,” “studies examining injuries or diseases,” and “studies on non-athlete samples.” The 29 remaining articles were checked for eligibility by reading the entire manuscript, which resulted in three more articles being excluded (i.e., were either neuroscience-based or examined a motor outcome rather than perceptual-cognitive performance). An overview of the complete selection process is presented in Fig. 1. In total, 26 articles were included in the review (see Table 1).

**Table 1:** Literature on the impact of physical exercise on perceptual-cognitive performances of athletes (*n =* 26)

Study (year)	Sport and Participants (*n*)	Exercise Load	Perceptual/Cognitive Task and Measurement	Testing Notes	Main Results	MMAT
Casanova et al. (2013) [26]	**Soccer Players** Experts: *n* = 8 *M =* 24.6 ± 3.9 Advanced: *n* = 8 *M =* 26.3 ± 2.9	**Specific** treadmill, intermittent exercise protocol	**Specific** ^a^ computer-based, anticipation, accuracy	during exercise, no rest condition, counterbalanced order	Reduced accuracy under fatigue for both groups	****
Cereatti et al. (2009) [36]	**Orienteers** Advanced: *n* = 12 *M =* 15.9 ± 1.4 Novices: *n* = 12 *M =* 15.6 ± 1.8	**General** cycle ergometer, moderate, 60 % HRR	**General** ^b^ computer-based, attentional task, Exp 1: focusing of attention at (para-) foveal locations Exp 2: peripheral visual space, RT, error rates	during exercise, rest condition, counterbalanced order	Exp 1: both groups improved RT during PE, orienteers improved more Exp 2: both groups improved RT during PE, no difference between groups	***
Collardeau et al. (2001) [28]	**Triathletes** Experts: *n* = 11 *M =* 26.5 ± 4.8	**Specific** treadmill, run at ventilatory threshold	**General** ^b^ computer-based, simple RT	before, during, after exercise, no counter-balanced order	Improvement in RT compared to rest after 40 min of PE, improvements from pre-exercise to after exercise	**
Davranche & Audiffren (2004) [37]	**Decision-Making Sport Athletes (handball, basketball, tennis, soccer)** Advanced: *n* = 16 *M =* 22.8 ± 2.5	**General** low (20 %) and moderate (50 %) ind. W_max,_cycle ergometer	**General** ^b^ computer-based, choice RT task, speed, accuracy	during exercise, rest condition, counterbalanced order	Improvements of RT (50 % compared to rest), no effect of PE on accuracy	****
Davranche et al. (2006) [38]	**Decisional Sport Athletes (Team Sport Players)** Advanced: *n =* 11 4 Female: *M =* 22 ± 2.0 7 Male: *M =* 25 ± 4.0	**General** cycle ergometer, moderate, 90 % of ind. ventilatory threshold power	**General** ^b^ computer-based, choice RT task, mean RT, decision errors	during exercise, rest condition, counterbalanced order	Faster RT during PE, no effect of PE on decision errors	***
Davranche et al. (2009) [39]	**Kayakers** Experts: *n* = 12 *M = n.r. (*14-35)	**Specific** Kayak ergometer, low (40 %) and moderate (75 %) ind. HR_max_	**General** ^a^ computer-based, Simon task, accuracy, RT	during exercise, rest condition, counterbalanced order	No effect on accuracy, RT better at 75 % compared to 40 %	****
Delignières et al. (1994) [40]	**Fencers** Experts: *n* = 20 *M =* 24.0 ± 8.3 Novices: *n* = 20 *M =* 23.3 ± 5.5	**General** cycle ergometer, low (20 %), moderate (40 %, 60 %), high (80 %) ind. W_max_	**General** ^b^ computer-based, 2- and 4-CRT tasks, speed, error rate	during exercise, rest condition, no counter-balanced order	Speed:	***
					Experts: improvents as PE increased, Novices: deterioration as PE increased, Error Rate: no differences	
Elsworthy et al. (2014) [25]	**Australian Football Players** Experts: *n* = 29 *M* = 32.4 ± 6.1	**Specific** intermittent real game analyses	**Specific** ^a^ decision-making, accuracy	during exercise, no rest condition, no counterbalanced order	No effect of PE on accuracy	****
Fontana et al. (2009) [44]	**Soccer Players** Advanced: *n* = 16 *M =* 21.1 ± 1.6 Novices: *n* = 16 *M =* 19.5 ± 1.1	**General** treadmill, rest, low (40 %), moderate (60 %, 80 %) ind. VO_2_max	**Specific** ^a^ video clips, decision-making task, speed, accuracy	during exercise, rest condition, counterbalanced order	Speed: improvements for both groups with increased PE intensity, PE does not affect accuracy	****
Hancock & McNaughton (1986)^1^[27]	**Orienteers** Advanced: *n* = 6 *M =* 27.0 ± 11	**Specific** treadmill, moderate, ind. anaerobic threshold	**Specific** ^a^ computer-based, slides of orienteering checkpoints + questions, correct/incorrect answers	during exercise, rest condition, counterbalanced order	PE-condition (= fatigue): decrease of correct answers	*
Hogervorst & Riedel (1996) [35]	**Triathletes, Cyclists** Advanced: *n* = 15 *M =* 24.9 ± 7.9	**Specific** cycle ergometer, moderate, 75 % ind. W_max_	**General** ^a^ computer-based, simple and 3 CRT, Stroop task	after exercise, rest condition (before exercise), no counter- balanced order	Speed: improvements after exercise for simple and 3 CRT task, Stroop task	**
Huertas et al. (2011) [32]	**Cyclists** Advanced *n* = 18 *M* = 17.0 ± 2.0	**Specific** cycle ergometer, moderate, 80 % and 90 % of lactate threshold	**General** ^a^ attentional task (including alerting, orienting, executive control), speed	after exercise, rest condition, counterbalanced order	Improvements under PE (90 % better than 80 %, both better than at rest)	****
Hüttermann & Memmert (2014) [34]	**Decisional Sport Athletes (Team Sport Players)** Advanced: *n* = 8 *M* = 24.88 ± 3.27 Novices *n* = 8 *M* = 26.00 ± 4.27	**General** cycle ergometer, moderate, 50 %, 60 %, 70 % HR_max_	**General** ^b^ computer-based, attentional breadth task, accuracy	during exercise, rest condition, counterbalanced order	Athletes: Improvements under PE, best results under 70 % HR_max_ Novices: Improvements under PE, bests results under 60 % HR_max_	***
Larkin et al. (2014) [46]	**Australian Football Players** Expert umpires: *n* = 15 *M =* 36.0 ± 13.5	**Specific** real games, high, competitive Australien football	**Specific** ^a^ video-based, decision-making, accuracy	post exercise, no rest condition, no counter-balanced order	Improvements in quarter 4 (compared to quarter 2 and 3)	****
Lemmink & Vjsscher (2005) [41]	**Soccer Players** Advanced: *n* = 16 *M =* 20.9 ± 2.0	**General** cycle ergometer, intermittent exercise	**General** ^b^ computer based, multiple choice RT task, speed, accuracy	Group 1: post exercise Group 2: rest	No differences between the groups	***
Llorens et al. (2015) [33]	**Triathletes** Advanced: *n* = 14 Novices: *n* = 13 *M =* 24 ± 3.0 (of both groups)	**Specific** cycle ergometer, high, maximal incremental effort	**General** ^a^ computer based, spatial attention task, RT	after exercise, rest condition, counterbalanced order	Improvements of RT for the advanced group under PE, No differences between the conditions for the novice group	****
McMorris & Graydon (1996) [45]	**Soccer Players** Advanced: *n* = 10 *M = n.r.* Novices: *n* = 10 *M = n.r.*	**General** cycle ergometer, rest, moderate (70 %), high (100 %) ind. W_max_	**Specific** ^b^ computer-based, decision-making task, accuracy, overall speed of decision, speed of decision for accurate responses	during exercise, rest condition, no counter-balanced order	Speed for accurate responses: improvements under PE, Overall speed: improvements only for advanced under PE, Accuracy: no effect of PE	***
McMorris & Graydon (1997) [20]	**Soccer Players** Exp 1: Advanced *n* = 12 *M =* 20.8 ± 1.34 Exp 2: Advanced *n* = 12 *M =* 20.8 ± 1.78	**General** cycle ergometer, rest, moderate (70 %), high (100 %) ind. W_max_	**Specific** ^a+b^ detection task/visual search, Exp 1: low complexity- 2c, Exp 2: high complexity-4c, speed of search, speed of decision, accuracy	during exercise, rest condition, no counter-balanced order	Exp 1: speed: improvements during maximal exercise, accuracy: no effect Exp 2: speed: improvements during PE compared to rest, accuracy better at 100 % than at rest	***
Mouelhi Guizani et al. (2006) [42]	**Fencers** Experts: *n* = 12 *M =* 19.1 ± 2.99 Novices: *n* = 12 *M =* 20.82 ± 3.97	**General** cycle ergometer, low (20 %), moderate (40 %, 60 %), high (80 %) ind. max. aerobic power	**General** ^b^ computer-based, simple and four choice RT task, RT, error rates	during exercise, rest condition, counterbalanced order	Fencers: shorter CRTs at 40 %, 60 % and 80 % P_max_compared to rest-Novices: no effect of PE	****
Pesce & Audiffren (2011) [29]	**Athletes from different Sports (Soccer Players, swimmers, gymnasts, rowers, orienteers, runners)** Advanced: Young Athletes: *n* = 53, *M* = n.r. (16-24) Older Athletes: *n* = 47 *M* = n.r. (65-74)	**General** cycle ergometer, moderate, 60 % HRR	**General** ^a+b^ computer-based, two reaction-time tasks (low and high cognitive demands), RT	during exercise, rest condition counterbalanced order	Low demanding task: no effects of PE, High demanding task:improvements under PE for all participants	***
Pesce et al. (2007a) [19]	**Orienteers** Experts: *n* = 12 *M* = 66.2 ± 4.7 Novices *n*=13 *M =* 66.3 ± 4.6	**General** cycle ergometer, moderate, 60 % HRR	**General** ^a+b^ computer-based, visual attention, Exp 1: low demands, Exp 2: high demands, RT, error rates	during exercise, rest condition, counterbalanced order	Accuracy and speed not influenced by PE for both groups and in both experiments	****
Pesce et al. (2007b) [43]	**Soccer Players** Experts: *n* = 24 *M =* 17.8 ± 0.8 Novices: *n* = 24 *M =* n.r., same age range	**General** cycle ergometiter, moderate, 60 % HRR	**General** ^a+b^ computer-based, visual attention, Exp 1: low demands, Exp 2: high demands, RT, error rates	during exercise, rest condition, counterbalanced order	Error rates: no effects on error rates in both Experiments Speed: Exp 1: no influence of PE on soccer players, improvements under PE for non-athletes, Exp 2: both groups: improvements under PE	****
Pesce et al. (2011) [30]	**Cyclists** Advanced: *n* = 16 Other endurance athletes: *n* = 16 Novices: *n* = 16 *M =* n.r. (60-80)	**Specific** cycle ergometer, moderate, 60%HRR	**General** ^b^ computer-based, visual attention, RT	during exercise, rest condition, counterbalanced order	Athletes faster under PE, no influence of PE on novices	****
Royal et al. (2006) [47]	**Water Polo Players** Experts: *n* = 14 *M =* 17.2 ± 0.5	**Specific** high, 4 sets of 8 repetitions of an 18 s max specific drill, progressively declining rest ratios (80-40-20-10s)	**Specific** ^a^ video-based, decision-making test, accuracy	after exercise, rest condition, counterbalanced order	Set 4 accuracy better than at the set 3,2,1 (but not sign higher than pre-exercise)	****
Tsorbatzoudis et al. (1998) [31]	**Cyclists** Experts: *n =* 12 Novices: *n* = 46 3 exercise groups (A,B,C): *n* = 12 in each group, 2 control groups, *n*=11, A + B: untrained students, C: cyclists, control group B + C	**Specific** cycle ergometer, A: high intensity exercise for 5 min, heart rate at 180-190 bpm, B + C: moderate intensity exercise for 30 min, heart rate 150-160 bpm	**General** ^b^ computer-based, simple RT task, Vienna Test System, RT	after exercise, rest condition, no counter-balanced order	All groups improved their RT in both tests after PE	***
Vickers & Williams (2007) [23]	**Biathletes** Experts: *n* = 10 *M* = n.r. (16-24)	**General** cycle ergometer, moderate (55 %, 70 %), high (85 %,100 %) ind. VO_2max_	**Specific** ^b^ biathlon shooting, visual attention, accuracy, gaze behavior - duration of quiet eye (QE)	after exercise, rest condition, counterbalanced order	Highest level of accuracy during 55 %, declined thereafter to the lowest level at 100 %, QE duration longer on hits than misses for 55 %, 70 %, 85 %; during 100 % it declined to half	****

*Abbreviations*: *PE*physical exercise, *n.r.*not reported, *ind.*individual, *RT*reaction time, *CRT*choice reaction time, *W*_*max*_maximum power output, *HR*_*max*_maximum heart rate, *VO*_*2max*_maximum oxygen uptake, ^a^working memory task, ^b^attention/perception task, *,**,***,**** MMAT score.

^1^The results from the study did not solely contain perceptual-cognitive tasks. First, slides of orienteering checkpoints were shown and afterwards participants had to answer questions concerning aspects of short-term memory, focus of attention, map interpretation, estimation or descriptive abilities. Results were reported for the amount of correct/incorrect answers as a whole, but not separately for the different aspects. Therefore, it cannot be concluded if and how much the physical exercise influenced the perceptual-cognitive tasks specifically.

### Categories

Results were analyzed with special consideration of the perceptual-cognitive task tested, the induced physical exercise, the time of testing, and the participants included in the study. In total, 707 participants from the 26 included articles were tested, ranging from 14–80 years of age. The identified articles examined a total of 194 expert, 307 advanced, and 206 novice athletes. Looking at the methodological quality of the individual studies, 23 articles reached a MMAT score [18] of three or four (75–100 % of criteria met). Thus, the overall methodological quality of the studies included in this systematic review was high.

#### Perceptual-Cognitive Task

Twenty-four studies used computer- or video-based tasks, with the only exceptions being Vickers and Williams [23] study which measured “quiet eye” durations in biathletes as a perceptual outcome (for more information and a review on quiet eye literature, see [24]) and the study by Elsworthy et al. [25] in which decision-making performance was measured in real-game situations. Most of the studies included either simple or choice reaction-time and attentional tasks in a general or specific setting (by e.g., showing sport-specific videos), with the exceptions being the studies by Vickers and Williams [23] and Casanova et al. [26] which investigated the accuracy of anticipation tasks, and Hancock and McNaughton [27] who focused on short-term memory performance.

An overview of results with a focus on the specificity of the perceptual-cognitive task tested can be seen in Table 2. From the 26 articles, 17 used a general task and nine used a specific task. Of the 17 studies that implemented a general task, six measured only reaction times [28–33], one measured only accuracy [34], and ten measured both reaction times and error rates [19, 35–43]. Fourteen of these studies revealed improvements for reaction times under acute physical exercise, while the study from Delignières et al. [40] and from Llorens et al. [33] only showed this for athlete groups. The studies from Pesce et al. [43], from Lemmink and Visscher [41] did not reveal a change in reaction times under the influence of physical exercise. Concerning the 11 studies that examined accuracy (i.e., error rates) in general perceptual-cognitive tasks, only Huttermann and Memmert [34], who did not induce any time pressure while performing an accuracy-based task, revealed improvements under physical exercise. All other studies looking at accuracy did not find any differences between conditions.

**Table 2:** Main results for speed and accuracy of the perceptual-cognitive performances based on the specificity (general or specific) of the perceptual-cognitive task

		+	o	−
general (*n* = 17)	speed (*n* = 15)	*n* = 14 [19, 28–33, 35–40, 42]	*n* = 2 [41, 43]	*n* = 1 [40]
	accuracy (*n* = 10)	*n* = 0	*n* = 10	*n* = 1
			[19, 35–43]	[34]
specific (*n* = 9)	speed (*n* = 3)	*n* = 3 [20, 44, 45]	*n* = 0	*n* = 0
	accuracy (*n* = 9)	*n* = 4 [20, 23, 46, 47]	*n* = 3 [25, 44, 45]	*n* = 2 [26, 27]

“+“ indicates an improvement of the perceptual-cognitive performance under physical exercise, “0“ no change and “-“ a deterioration

Out of the nine studies using a specific perceptual-cognitive task, three considered reaction times and error rates [20, 44, 45], five measured only accuracy [25–27, 46, 47], and the study by Vickers and Williams [23] measured “quiet eye” durations in biathletes as a perceptual outcome. The three studies considering reaction times found an improvement under acute physical exercise. Studies of accuracy in specific perceptual-cognitive tasks displayed variable results: four studies revealed improvements [20, 23, 46, 47], two studies found deteriorations [26, 27], and three studies showed no effect [25, 44, 45].

Interpreting the results focusing on the types of tasks, eleven studies used a working memory task [23, 26, 27, 29, 32, 35, 39, 44–47], 11 studies attention/perception tasks [28, 30, 31, 33, 34, 36–38, 40–42], and four included both types of tasks in two experiments included in the studies [19, 20, 29, 43]. The results for speed show that seven studies find improvements under physical exercise in working memory tasks [19, 20, 29, 32, 35, 39, 44] and 11 studies in attention/perception tasks [20, 28, 30, 31, 33, 36–38, 40–42]. No differences were found in one study in a working memory task [43] and in two studies using an attention/perception task [19, 43]. For accuracy in working memory tasks, five studies showed improvements under physical exercise [20, 23, 45–47], five found no changes [19, 25, 35, 39, 44], and three found deteriorations [26, 27, 45]. For accuracy in attention/perception tasks, two studies found improvements [34, 41] and six studies found no changes of perceptual-cognitive performances under physical exercise [19, 20, 28, 36–38].

#### Physical Exercise

An overview of results with a focus on the acute physical exercise induced can be seen in Table 3. From the 26 articles included in this review, 14 implemented a general physical exercise while 12 implemented a specific one. From the studies using a general physical exercise, six induced a moderate intensity [19, 29, 34, 36, 38, 43], two studies tested under two different conditions (a low and a moderate intensity condition) [37, 44], three studies under two conditions (moderate and high intensity) [20, 23, 45], and two studies measured all three levels of exercise intensity in different conditions [40, 42]. Only one study [41] used an intermittent exercise, alternating a high-intensity exercise (40s) with a low-intensity exercise (20s) for 8 min in total. Of the 12 studies that implemented specific physical exercises, two studies induced an intermittent exercise [25, 26]. One of these studies included two identical periods of 52 min each, each containing five low- and two high-intensity blocks [26] and the other measured performance in real-game situations, meaning that low-, moderate-, and high-intensity demands occurred [25]. In addition, five studies induced a moderate intensity exercise [27, 28, 30, 32, 35], three high intensities [33, 46, 47], one both low and moderate intensity [39], and one both moderate and high intensity in different conditions [31].

**Table 3:** Results for speed and accuracy of the perceptual-cognitive performances based on the physical exercise induced

phys ex	intensity	accuracy			speed		
		+	o	−	+	o	−
general (*n* = 14)	low *n* = 4	*n* = 0	*n* = 2 [40, 44]	*n* = 0	*n* = 0	*n* = 3 [37, 42, 44]	*n* = 0
	moderate *n* = 13	*n* = 2 [23, 34]	*n* = 9 [19, 20, 36–39, 43–45]	*n* = 0	*n* = 10 [19, 20, 29, 36–38, 40, 42, 44, 45]	*n* = 1 [43]	*n* = 0
	high *n* = 5	*n* = 0	*n* = 3 [20, 40, 45]	*n* = 1 [23]	*n* = 4 [20, 40, 42, 45]	*n* = 0	*n* = 0
	intermittent *n* = 1	*n* = 1 [41]	*n* = 0	*n* = 0	*n* = 1 [41]	*n* = 0	*n* = 0
specific (*n* = 12)	low *n* = 1	*n* = 0	*n* = 0	*n* = 0	*n* = 0	*n* = 1 [39]	*n* = 0
	moderate *n* = 7	*n* = 0	*n* = 1 [39]	*n* = 1 [27]	*n* = 5 [28, 30–32, 35, 39]	*n* = 0	*n* = 0
	high *n* = 4	*n* = 1 [47]	*n* = 1	*n* = 0	*n* = 3 [31, 33, 46]	*n* = 0	*n* = 0
	intermittent *n* = 2	*n* = 0	*n* = 1 [25]	*n* = 1 [26]	*n* = 0	*n* = 0	*n* = 0

“+“ indicates an improvement of the perceptual-cognitive performance under physical exercise, “0“ no change and “-“ a deterioration

Results for the general and specific acute physical exercise loads did not differ. Under both types, the majority of the studies reported no effect on accuracy. The number of studies showing a negative effect [23, 26, 27] and those showing a positive effect [34, 41, 47] was equal. Differences between the perceptual-cognitive performances were observed between different exercise intensities. While no study revealed an effect for low physical exercise, the majority of studies found positive effects for moderate physical exercise on reaction times [19, 20, 28–32, 35–40, 42, 44, 45], with the exception of the study from Pesce et al. [43] which found no effect. For accuracy, only one study showed a negative effect of moderate exercise on perceptual-cognitive performances [27] and one study found a positive effect [34], while most of the studies did not find an effect [19, 20, 36–39, 43–45]. Similar results were found with high-intensity exercise. The majority of studies showed positive effects on speed [20, 31, 33, 40, 42, 45, 46], but no effect on accuracy [20, 40, 45]. The exceptions were Royal et al. [47] who found a positive effect of a high physical exercise on accuracy in perceptual-cognitive tasks, and Vickers and Williams [23], who found a negative effect under 100 % VO_2max_.

#### Time of Testing

There are different ways of measuring perceptual-cognitive tasks in combination with physical exercise. For example, the task can either be tested during or after the physical exercise has taken place. Collardeau et al. [28] conducted both measurements, while seven studies tested the perceptual-cognitive performance after completing the physical exercise [23, 31, 33, 35, 41, 46, 47]. The remaining 18 studies had participants perform the perceptual-cognitive task during the physical exercise [19, 20, 25–27, 29, 30, 32, 34, 36–40, 42–45]. Despite these different methodological approaches, there were no obvious differences in the perceptual-cognitive performances depending on the time of testing.

#### Participants

From the 26 articles, 13 considered athletes from individual sports, such as triathlon [28, 33, 35], biathlon [23], cycling [30–32], orienteering [27, 36, 43], kayak [39], or fencing [40, 42]. Of the 12 studies examining team sports, six looked at soccer [19, 20, 26, 41, 44, 45], three at athletes from different team sports (e.g., handball, basketball, soccer) [34, 37, 38], two at American football [25, 46], and one at water polo [47]. Pesce and Audiffren [29] examined a mixed group of participants from sports such as swimming, rowing, gymnastics, or soccer. They found no performance differences between those competing in individual or team sports. The majority of studies from both classifications (individual and team) showed a positive effect of acute physical exercise on reaction times [19, 20, 23, 28–33, 35–40, 42, 44, 45] but no effect on accuracy [19, 20, 25, 37–40, 42–45].

Fourteen studies examined athletes and compared the impact of the physical exercise under different conditions, such as rest-physical exercise or different intensities of physical exercise [20, 23, 25–29, 32, 35, 37–39, 46, 47]. Lemmink and Visscher [41] divided soccer players equally into an exercise group and a non-exercise group. Eleven studies contained a novice or control group in addition to a group of athletes and tested both groups under all conditions [19, 30, 31, 33, 34, 36, 40, 42–45]. Seven of these studies suggested that expert and advanced athletes are influenced by acute physical exercise differently than novices, with the higher performing athletes improving their performances under physical exercise while novices did not or less [30, 33, 34, 36, 40, 42, 45]. The other four studies reported no differences between athletes and non-athletes, which suggests that no study showed improvements for non-athletes only. Thus, the overarching finding shows a tendency of athletes to benefit more from acute physical exercise than novices.

## Discussion

The aim of this review was to provide a more complete understanding of the impact of acute physical exercise on perceptual-cognitive performances of athletes. As a whole, the majority of studies included in this systematic review suggested that athletes’ perceptual-cognitive performances in speed-related tasks are improved by moderate- and high-intensity physical exercise while accuracy is not influenced. Furthermore, results indicated that athletes’ perceptual-cognitive performances are more positively influenced by physical exercise than those of non-athletes.

However, looking at the specificity of the perceptual-cognitive task, results of this systematic review suggest that athletes’ reaction times improved for both general and specific tasks under acute physical exercise. However, acute physical exercise seems to influence accuracy differently based on whether a general or specific task was used. Eight of the 11 studies showed that physical load did not influence athletes’ accuracy in general tasks, which reflects prior research in general experimental setups where participants’ reaction times improved while accuracy failed to change under physical exercise (e.g., [4]). This might be explained by either a ceiling effect or by the nature of the tasks that were used [10]. As McMorris and Hale [4] indicated, some tasks (e.g., Simon task or choice reaction-time tasks) primarily focus on testing speed of processing. Accuracy measurements are mainly meant to control participants’ focus on solving the tasks and to ensure that no speed-accuracy trade-off occurs [4, 10]. However, results for accuracy related to specific tasks were more inconsistent, with four studies showing improvements [20, 23, 46, 47], two deteriorations [26, 27], and three with no effect [25, 44, 45]. Looking at the types of tasks that were used within the nine studies that included specific perceptual-cognitive tasks, no pattern in the various tests was found. For example, decision-making tasks were used in all of the three groups of results (improvements, no differences, or deteriorations). Accordingly, there was not enough consistency in the outcomes and tests of the studies reviewed as a whole, which prevents this review from establishing any clear conclusions on the effect of acute physical exercise on specific perceptual-cognitive performances of athletes. Since the sample sizes, range of cognitive tasks, and number of studies are small, replication of these results is important. Moreover, future research should further investigate how physical exercise influences athletes’ performances in accuracy using a broader range of specific perceptual-cognitive tasks. Results for the type of the task (working memory task or attention/perception task) do not seem to provide a structured pattern. Therefore, no clear conclusion can be drawn whether *athletes’* perceptual-cognitive performances are also influenced differently depending on the type of the task tested, as recent research indicates (cf. [3, 4, 10]). A possible reason for the lack of differences between these task types might be the complexity of the tasks that were used in the studies. Although working memory tasks were examined in several studies included in this systematic review (e.g., [19, 43, 44]), it is possible that the tasks were still not complex enough, as the tasks used were less complex than, for example, tasks that include planning, abstract thinking or cognitive flexibility [4]. However, looking at the specificity of the task (general vs. specific), athletes’ accuracy seems to be influenced differently depending on the specificity of the perceptual-cognitive task, which is in line with prior research suggesting expertise is highly specific and may not be captured using general tests (e.g., [48, 49]). Given these limitations regarding how physical exercise influences athletes’ perceptual-cognitive performances in specific tasks, a fruitful area for further research would be to address whether specific physical exercise load influences athletes’ *accuracy* in specific perceptual-cognitive tasks, since this seems to be different than in general tests.

Results regarding exercise intensity suggested no effect of low intensity on perceptual-cognitive task performance, while moderate to high intensities seem to increase speed but not accuracy. This is partly in line with prior research on the impact of acute physical exercise on general perceptual or cognitive tasks. Prior research suggests an inverted U-relationship, with moderate physical exercise intensity having a positive effect on perceptual-cognitive performance, while high exercise intensity having a negative effect ([9], e.g., [50]). However, this inverted U-relationship has been criticized and recent research emphasizes that the effects of physical exercise on cognitive tasks are especially task-type dependent (e.g., [10]). As McMorris et al. [10] suggested, moderate physical exercise seems to influence working memory and attention/perception tasks positively while high-intensity exercise improves attention/perception tasks but impairs working memory tasks. The results of this review note a positive effect for high exercise intensities for both types of tasks, reinforcing the conclusion that athletes respond differently to acute physical exercise than non-athletes. This assumption is also supported by several studies that included athlete and novice groups in their experiments [30, 33, 34, 36, 40, 42, 45]. These studies showed that athletes are more positively influenced by acute exercise than non-athletes, which might be explained by athletes’ familiarity with exposure to high physiological stress accompanied by high physical and mental loads. However, it is uncertain whether the level of expertise of the athlete plays a role in this context, since the examined articles do not provide very detailed athlete experience data. While well-described for the purpose of the individual studies, the lack of a standardized classification of athletes across all of the studies limits the generalizability of certain conclusions about the role of the exact level of expertise in this context. Emphasizing how important a detailed definition of expertise is, Baker et al. [51] proposed a taxonomy for researchers in skill acquisition and expertise. As such, future research should aim to address how physical exercise influences perceptual-cognitive performances of athletes at different levels of skill and classify these athletes using a more sensitive classification system.

**Fig. 2:**
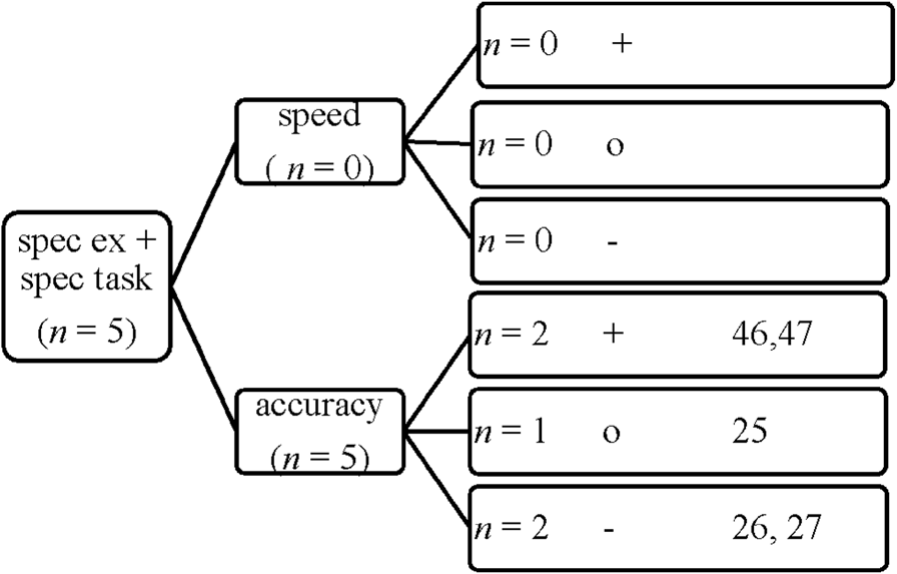
Overview of results from studies inducing a specific physical exercise and testing a specific perceptual-cognitive task. “+” indicates an improvement of the perceptual-cognitive performance under physical exercise, “0” no change and “-” a deterioration

Five studies induced a specific acute physical exercise in combination with a specific perceptual-cognitive task. Interestingly, as can be seen in Fig. 2, all of the five studies looked at *accura*cy of perceptual-cognitive tasks and the results were inconsistent (none examined speed). While two studies found reductions in accuracy with enhanced fatigue in an anticipation [26] or memory [27] task, two studies found improvements in decision-making tasks [46, 47], and one study found no effect in a decision-making task [25]. This might be explained by the different types of perceptual-cognitive tasks used; however, none of the studies included in this review looked at speed when including a specific exercise and a specific task. Furthermore, data on accuracy are also relatively limited. This gap of expertise research should be addressed more specifically through examining the impact of physical exercise on perceptual-cognitive performances of athletes using more rigorous methods that measure speed and accuracy and induce a highly specific physical exercise under which a sport-specific task has to be performed.

Collectively, these results highlight the various elements of specificity as they relate to perceptual-cognitive expertise. The results concerning skill-based differences, with suggesting skilled athletes profiting more from acute physical exercise than novices, is particularly noteworthy. The need to couple the specific perceptual-cognitive demands of the task with motor execution (i.e., perception-action coupling) has been widely supported in motor learning and motor control research [52]. According to Gibson [53], perception and action are highly dependent on each other, and changing or manipulating one automatically influences the other [52, 53]. In the context of sport, this assumption has been supported by many studies inducing tasks where perception and action were directly coupled (e.g., [7, 54, 55]). The results of a meta-analytical investigation on effects of exercise on cognition highlight that “when perception and action are combined, the complexity of the interaction induces different effects to when cognition is detached from motor performance” ([7], p. 180). If we extend this frame of mind to the results of this systematic review, it is possible that experts, through their extensive domain-specific experience, are more accustomed to the specific coupling of acute physical exercise and perceptual-cognitive tasks. Because of their extensive practice and competitions in their field of expertise, they can handle performing a perceptual-cognitive task while under high physiological stress better than less experienced participants.

Results of the current review suggest other specificity-related parameters may also underpin the complex phenomenon of perceptual expertise. In particular, intensity-related variables may be especially important (e.g., the coupling of perception-action and intensity). While this review focused on intensity in regard to physiological stress, it is likely that intensity in this context reflects a broad term covering a range of factors related to the specificity of the measurement environment (e.g., physical load, the combination of physical and cognitive load, performing under time pressure, as well as performance-related emotions such as nervousness and competition anxiety). Prior research in sport expertise and applied sport psychology revealed that experts are superior to non-experts in several different domains of expertise, such as controlling and regulating emotions effectively (for an overview see [56]). The term *perception-action-intensity coupling* may help to explain expertise differences found in this systematic review as well as in other domains. Although this notion has some intuitive appeal, additional work is clearly necessary to determine the validity of this argument.

This systematic review focused on perceptual-cognitive performance, which is very important when athletes actually execute their specific sport. However, to fully capture, explain, and understand the impact of physical exercise on perceptual-cognitive expertise, it is necessary that future research also considers the underlying mechanisms. Several authors have looked at the neurophysiological basis of perceptual-cognitive performance and have shown that catecholamins in the brain lead to different levels of arousal when under physical exercise, which influences the performances of perceptual-cognitive tasks [4, 7, 10]. Future research should extend this work by examining athletes of different levels of expertise.

### Limitations of the Current Review

The current review provides an important synthesis of how acute exercise loads affect perceptual-cognitive performance; however, there were several notable limitations. One is directly connected to the specificity of perceptual-cognitive expertise. Clearly, specificity is important in the context of expertise; however, we had to create broad categories in order to adequately summarize and combine results to provide an overview of the existing literature on the impact of different acute physical exercises on perceptual-cognitive performances of athletes. Although we believe this was a reasonable approach to examining the research questions addressed in this review, this may have eliminated some specific results of individual studies. It is possible that some of these detailed results that were not highlighted because of the broad categories we had to create might turn out to be important once more research in this area has been conducted. For example, the methodology applied in this review classified the physical exercise intensity induced using *W*_max_, VO_2max_, or HR_max_. For other given values of intensity (e.g., heart rate reserve), physical exercise was compared to the studies that provided data on *W*_max_, VO_2max_, or HR_max_ and the studies were accordingly classified into one of the groups. As McMorris and Hale [4] emphasized, this strategy contains a component of subjectivity.

Second, it was difficult to account for the different methodologies of the 25 articles included in this review. For example, not all of the studies tested the different exercise and rest conditions in a counterbalanced order (e.g., [20, 35, 44–46]). Therefore, it is not clear whether the found changes in perceptual-cognitive performances occurred due to the impact of the physical exercise induced or because of, for example, learning effects or familiarization with the task. Moreover, some studies included different exercise intensity conditions but no rest condition [25, 26, 46]. These studies, therefore, give an insight in how perceptual-cognitive performances change under different exercise intensities but do not allow us to draw any conclusions about a comparison to the performance at rest. In addition to the different methodologies applied, care should be taken when comparing and summarizing results from the different studies because of the different sample sizes and power effects across the studies. Since both are important factors when evaluating the validity and significance of results (e.g., [57]), it should be noted that there was considerable variability in the sample sizes between the different studies included in this review. For example, Hancock and McNaughton [27] examined six participants and Vickers and Williams [23] examined ten participants, whereas Pesce and Audiffren [29] included 100 participants in their study. All of the other studies had sample sizes within this range (i.e., 6 to 100 participants).

Third, this review contained a relatively large age range (between 14 and 80 years) of participants. Since this review did not focus on differences between age groups, this factor was not considered. However, the impact of physical exercise on perceptual-cognitive performances across age groups is an interesting field of further research.

Fourth, there was a disproportion in the types of sports presented in the 26 articles included in this review. Although the distribution of individual (13 studies) and team sports (12 studies) was similar (one study included athletes from different types of sports), a closer look at the sports within these categories revealed a disproportionate focus on soccer in the team sport category (six of 12 studies). Future research should therefore look at the impact of physical exercise on specific perceptual-cognitive tasks across a more diverse range of sports. Looking at this field of research from different perspectives and different sports may provide important information for the field of expertise and help apply this knowledge to specific sport contexts.

Finally, one of the inclusion criteria in this systematic review was that the articles had to be written in English. Since English is the most common language in this field of research and most important articles are (also) published in English, this is in adequate method for conducting a systematic review. However, it is possible that important studies relevant to this review may exist in other languages.

## Conclusions

Results of this review suggest acute physical exercise has an influence on the perceptual-cognitive performance of athletes. Moderate and high physical exercise improves speed in perceptual-cognitive tasks and does not influence accuracy in the majority of studies. Athletes seem to be more positively influenced by acute physical exercise than non-athletes. However, specificity plays an important role in the science of expertise, both in understanding the limitations of perceptual-cognitive skill and in designing valid and reliable tasks to measure these qualities. As a result, future research should examine these effects using more specific testing. For example, inducing an intermittent interval exercise for a particular team sport and testing specific perceptual-cognitive performances under real-game situations, whether during or in between competition, would be valuable for future researchers and sport practitioners to explore. While this review sums up the main findings of the impact of acute physical exercise on perceptual-cognitive expertise, care should be taken when making general conclusions. Since many factors influence perceptual-cognitive expertise and many factors interact with one another [10], it is unclear how much each single factor influences the outcome (e.g., is it the type of the physical exercise itself or a combination of the timing of the testing and the specific participants tested?). This leads to a clear need for future research that is highly precise and specific, such as when defining the expertise level of participants, choosing a specific physical exercise and a specific perceptual-cognitive task.
